# Ontario Veterinary Association

**Published:** 1900-02

**Authors:** C. H. Sweetapple

**Affiliations:** Secretary


### ONTARIO VETERINARY ASSOCIATION.

The annual meeting was held in the Veterinary College, Toronto, Canada, on Friday, December 22, 1899. The meeting was opened at 11.15 A.M. In the absence of Mr. S. Sisson, the President, the First Vice-President, Mr. W. J. Wilson, of London, took the chair, and after a few well-chosen remarks he called for the reading of the minutes of the last meeting, which were read and confirmed. New members were then proposed and accepted, and some routine business was transacted. The meeting then adjourned for lunch.

On opening the meeting after lunch the chairman called as the first order of business for the Secretary's, Treasurer's, and Registrar's reports. A very large mass of correspondence was reported, among the most important being numerous letters from all over the province of Ontario relating to measures that were being adopted for endeavoring to procure better legal protection for our profession. Also correspondence relating to the prosecution and conviction of one T. Johnson for illegally advertising and practising as a veterinary surgeon. He also reported fourteen graduates having registered since the last annual meeting. The Auditor's report showed the finances to be in a good condition.

The report of the committee appointed in connection with the efforts made in endeavoring to procure better legal protection for our profession was discussed, and it was shown that, notwithstanding a clause was inserted providing that all persons who had been practising veterinary surgery continuously in one place for fifteen years should be entitled to register, when it came before the Legal Committee for discussion it was found that a majority of that committee were opposed to the bill. The chairman of that committee, the Hon. J. M. Gibson, suggested that in consequence of the rather serious opposition the bill be withdrawn for this year, and if thought advisable introduced again at the next session of the Legislature when he thought it would likely be carried. After considerable discussion it was resolved that the bill should be left in the hands of Mr. German, M. P., to endeavor to carry it at the coming session of the Legislature, and that the old committee, consisting of Professor A. Smith, Mr. C. Elliott, Mr. Hutton, and the Secretary should be empowered to act in endeavoring to get it passed.

The Secretary read a letter from Mr. Giffen, V.S., Secretary of the Michigan State Veterinary Medical Association, giving a most cordial invitation to the members of the Ontario Veterinary Association to join with the Ohio and Michigan Associations in holding a joint meeting in Detroit, Mich., on September 5th next. All the members were requested to attend, and the Secretary was instructed to acknowledge the receipt of Mr. Giffen's cordial invitation.

The Secretary was instructed to send a letter of sympathy and condolence to the widow and family of the late Mr. J. H. Wilson, V.S., an old and valued member of this Association, in the loss they have sustained by the death of our much esteemed and respected confrere.

The Chairman then called on any members present to describe any cases of special interest which they have recently met with in their respective practices.

Mr. John Wende, V.S., of Buffalo, in response, described an outbreak of rabies which was quite extensive about Buffalo and the surrounding country, dogs, horses, cattle, and hogs having been affected. He said that 75 per cent. of the dogs had the disease in what was called the dumb form. He gave an interesting account of his inoculation experiments. He also said that there was no law in his locality to compel the quarantine of dogs.

Mr. J. H. Tennant, V.S., of London, gave an interesting account of his results in adopting Schmidt's treatment for parturient apoplexy. The treatment was surprisingly successful.

Mr. W. J. Wilson, V.S., of London, spoke of the good results he had seen in the treatment of deep-seated fibrous tumors by injecting into the tumors with a hypodermatic syringe a mixture of hydrochloric acid and pepsin.

Mr. John Wende exhibited an ingenious tube through which to insert the catheter in the cow.

All these subjects were extremely interesting, and many instructive remarks were elicited during the discussions that ensued.

It was resolved that the sum of twenty-five dollars be appropriated for a medal to be competed for by the graduating class at the next spring examinations.

The following officers were elected for the ensuing year: President, Mr. W. J. Wilson; First Vice-President, Mr. H. S. Wende; Second Vice-President, Mr. J. H. Tennant; Secretary and Treasurer, Mr. C. H. Sweetapple. Directors: Messrs. D. H. McMurtry, J. H. George, W. Steele, J. Wagner, W. Lawson, F. G. Hutton, W. Shillinglaw, and F. J. Gallanough. Auditors: Messrs. C. Elliott and J. D. O'Neal. Delegates to the Industrial Fair Association, Toronto: Professor A. Smith and Mr. W. J. Wilson. Delegates to the Western Fair Association, London: Messrs. J. D. O'Neal and J. H. Tennant.

C. H. Sweetapple,
Secretary.

